# Some Anomalies of the Circulatory Organs

**Published:** 1882-04

**Authors:** 


SOME ANOMALIES OF THE CIRCULATORY ORGANS.—In a cow which died from an internal hæmorrhage it was found that a needle had passed from the second stomach into the arch of the aorta. An English stallion, eight years old, during life had been noticed to have a very slow pulse, and to be subject at times to convulsions (angina pectoris ?). Post-mortem showed a marked eccentric hypertrophy of the right ventricle, and concentric hypertrophy of the left ventricle. A cow during life had a noticeable venous pulsation in the neck, and a large, firm, constant dilatation of the jugular vein. Post-mortem revealed a very great narrowing of the anterior vina cava, with thickening of the walls.

Hæmorrhoidal bleedings are occasionally observed in the cow and horse. In one case the bleeding veins were tied in a horse. Soon after, a bloody sweat exuded from the skin. The same bloody sweat has been observed in cows, without there being any apparent lesion of the skin.

Among the oxen used by the peasants of the Black Forest some curious cases of dyspnoea exist. The cause is the use of a very primitive form of yoke. This consists of a broad stick, which is tied across the breast of the ox. Thongs by which the plow is dragged are attached to either end. The stick presses on the trachea and causes a stenosis or narrowing of that tube.

